# Effects of Co-Application of γ-Polyglutamic Acid and Chemical Fertilizer on Rhizosphere Microbial Community Structure and Function of Cotton at Different Growth Stages in an Arid Cotton Field

**DOI:** 10.3390/microorganisms14091905

**Published:** 2026-08-28

**Authors:** Mingxuan Che, Jingbo Zhang, Kunduziayi Kudelaiti, Jiajun Zhang, Yunhao Liusui, Zhengwu Dong

**Affiliations:** 1 College of Life Sciences, Xinjiang Normal University, Urumqi 830017, China; 18290783479@163.com (M.C.); 18910445207@163.com (J.Z.); kunduzay386@163.com (K.K.); n20050720zjj@163.com (J.Z.); 2 Xinjiang Key Laboratory of Special Species Conservation and Regulatory Biology, Xinjiang Normal University, Urumqi 830017, China; 3 Key Laboratory of Special Environment Biodiversity Application and Regulation in Xinjiang, Xinjiang Normal University, Urumqi 830017, China; 4 College of Future Energy and Environmental Engineering, Xinjiang Normal University, Urumqi 830017, China

**Keywords:** arid region, cotton field, biostimulant, γ-polyglutamic acid, metagenomic sequencing technology, rhizosphere microbial community

## Abstract

Long-term excessive nitrogen application in arid cotton fields increases nitrate leaching risk during fallow and disrupts rhizosphere microecology. To clarify the cross-growth-stage regulatory effects of the biostimulant γ-polyglutamic acid (γ-PGA) combined with chemical fertilizer on rhizosphere microbial communities, we compared chemical fertilizer alone (NK) and γ-PGA plus chemical fertilizer (GT) using rhizosphere soils collected at boll-setting (August) and fallow (October), with physicochemical measurements and metagenomic sequencing technology. At boll-setting, GT lowered pH by 0.74 units compared with NK and increased NH_4_^+^-N, NO_3_^−^-N, and TN by 339.3%, 491.4%, and 23.0%, respectively. By fallow, GT increased TOC by 70.6% and maintained NH_4_^+^-N at 18.38 mg/kg, while NK accumulated 66.85 mg/kg NO_3_^−^-N. GT buffered post-harvest fungal community disturbance (Shannon: GT 4.06 vs. NK 2.80) and shifted bacterial communities toward oligotrophic taxa and archaea toward ammonium-preferring taxa. A metagenomic LEfSe analysis showed that GT was enriched in functional genes related to [Q]: Secondary metabolite biosynthesis, transport and catabolism, [T]: Signal transduction mechanisms, and [V]: Defense mechanisms, indicating a shift from resource acquisition to conservative maintenance. Mantel tests revealed that microbial functional profiles showed the strongest association with NH_4_^+^-N (r = 0.828 in August, r = 0.883 in October, *p* < 0.001). Thus, γ-PGA with chemical fertilizer stabilizes fallow rhizosphere microbial communities, reduces nutrient leaching, and promotes carbon–nitrogen co-retention.

## 1. Introduction

Cotton (*Gossypium hirsutum* L.), as a major source of natural fiber, is a cornerstone of the global textile industry. Global raw cotton production is projected to reach 29.5 million tonnes by 2034 [1]. To meet this demand, intensive agriculture often relies on the excessive application of synthetic nitrogen, phosphorus, and potassium (NPK) fertilizers. Long-term imbalanced nutrient inputs reduce nutrient use efficiency (NUE) and drive the accumulation of soluble salts in the rhizosphere [2,3]. Such soil stress factors not only impair root hydraulic conductivity and inhibit plant growth [4,5], but also profoundly alter the rhizosphere microenvironment. This disruption imposes selective pressure on beneficial microbial communities responsible for organic matter decomposition and nutrient cycling, thereby jeopardizing the long-term sustainability of agroecosystems [6]. Addressing these nutrient and salinity constraints is particularly important in arid and semi-arid agroecosystems, which are characterized by inherent water scarcity, high evapotranspiration, and fragile soil structure [7]. The Xinjiang Uygur Autonomous Region is a typical example of such environments, and optimizing its nutrient management can provide valuable reference for agriculture in arid regions worldwide [8]. Therefore, identifying novel biostimulants capable of improving soil health is currently a research hotspot.

γ-Polyglutamic acid (γ-PGA) is a biodegradable anionic heteropolymer synthesized by microbial fermentation, with excellent agricultural biostimulant activity [9,10]. Its agronomic value is mainly reflected in enhancing soil water retention, alleviating ion stress, and optimizing soil structure [11,12,13,14]. Specifically, γ-PGA can significantly improve soil water-holding capacity owing to its excellent hygroscopicity [11]. The abundant free carboxyl groups along its molecular chain can effectively chelate soil cations, thereby alleviating ion toxicity under saline–alkali stress [12]; meanwhile, the application of γ-PGA can promote the formation of water-stable macroaggregates, physically protect organic carbon, and synergistically improve crop yield and fiber quality [13,14]. These targeted improvements in the rhizosphere physicochemical environment not only directly benefit crop growth, but also impose novel habitat filtering pressures on soil microorganisms.

Soil microorganisms, as the core drivers of soil nutrient cycling, undergo significant community restructuring in response to targeted improvements in the soil physicochemical environment, potentially driving changes in associated ecosystem functions [15,16]. In fact, short-term rhizosphere microbial responses induced by γ-PGA have been repeatedly confirmed at the seedling stage: Yin et al. applied γ-PGA fermentation broth to maize pot experiments at the three-leaf stage and found that rhizosphere bacterial and fungal community compositions were significantly altered within 30 days, with the enrichment of plant growth-promoting bacteria such as *Bacillus* and *Pseudomonas* [17]; Ma et al. observed increased abundances of rhizosphere PGPR such as *Azospirillum* and *Streptomyces* accompanied by significantly enhanced urease activity only 7 days after γ-PGA application to maize roots [18]; and their team also detected selective enrichment of rhizosphere *Actinobacteria* and *Alphaproteobacteria* after treating two-leaf-stage maize with 50 mg·L^−1^ γ-PGA under severe drought stress for 30 days [19]. These studies have demonstrated that γ-PGA possesses promising plant biostimulatory effects; however, most have focused on a single growth stage, with few reports spanning the entire growing season. The aforementioned studies remain largely limited to short-term observations within a single growth stage, and systematic research on how γ-PGA drives the assembly of rhizosphere microbial communities across different growth stages over time is still lacking.

In recent years, with the rapid development of molecular biology techniques, metagenomic sequencing technology, which extracts DNA directly from soil, has become one of the most influential tools for studying the soil microbial environment, enabling the in-depth exploration of microbial biodiversity, community structure, and functional potential [20]. In this study, metagenomic sequencing technology was used as the core approach, combined with soil physicochemical analyses, to conduct field experiments in the arid region of Xinjiang. The rhizosphere microbial communities and soil environmental characteristics of cotton were systematically monitored at two key growth stages: August (boll-setting stage, the most vigorous growth phase) and October (post-harvest fallow period). This study aimed to (1) elucidate the effects of γ-PGA on the rhizosphere soil physicochemical properties of cotton at different growth stages, and (2) decipher the assembly processes of rhizosphere microbial communities and the mechanisms of functional potential reorganization under the reshaping of the soil physicochemical environment. This study is expected to provide a theoretical basis for improving sustainable soil nutrient management, thereby contributing to the sustainable development of agricultural production.

## 2. Materials and Methods

### 2.1. Experimental Site and Soil

The field experiment was conducted at the experimental field of Wenquan Campus, Xinjiang Normal University, Urumqi, Xinjiang Uygur Autonomous Region, China (44.96° N, 80.95° E). The region is characterized by a typical mid-temperate continental arid climate, with a long-term mean annual temperature of 7.7 °C, mean annual precipitation of 310 mm (concentrated mainly in summer), annual evaporation of 2570 mm, and an average frost-free period of 156 days [21]. The soil at the experimental site is a loam with the following properties: organic matter 13.2 g/kg, alkali-hydrolyzable nitrogen 63.25 mg/kg, available phosphorus 22.5 mg/kg, available potassium 210.3 mg/kg, and pH 7.61.

### 2.2. Experimental Design

The cotton cultivar used in this study was “Xinluzao 80,” bred by the Shihezi Academy of Agricultural Sciences. The fertilizers applied included a compound NPK fertilizer (N-P_2_O_5_-K_2_O = 15-15-15, total nutrients ≥ 45%) and a pure γ-polyglutamic acid (γ-PGA) granular formulation (γ-PGA content ≥ 10%, containing N 0.15%, P_2_O_5_ 0.11%, and K_2_O 0.41%), both provided by Xinjiang Huier Agricultural Group Co., Ltd (Changji, China). The experiment comprised two treatments: chemical fertilizer alone (NK) and chemical fertilizer combined with γ-PGA granules (GT), arranged in a randomized complete block design with three replicates per treatment. Each plot measured 2 m × 5 m, and adjacent plots were separated by 1 m guard rows. In the NK treatment, compound fertilizer was applied at 285 kg ha^−1^, supplying 42.75 kg ha^−1^ each of N, P_2_O_5_, and K_2_O. In the GT treatment, γ-PGA granules were additionally applied at 4.5 kg ha^−1^ on top of the same compound fertilizer rate, corresponding to an actual γ-PGA input of 450 g ha^−1^. The nutrient increments introduced by the γ-PGA granules (N 6.75 g ha^−1^, P_2_O_5_ 4.95 g ha^−1^, and K_2_O 18.45 g ha^−1^) were calculated to be negligible and therefore did not interfere with the intended nutrient supply.

Cotton was sown on 15 May 2023 under plastic film mulching. The mulch film was a conventional non-degradable white polyethylene (PE) film (thickness: 0.010 mm) produced by Xinjiang Tianye Group. Each film covered two rows, with a row spacing of 50 cm and a planting density of 222,000 plants ha^−1^. Throughout the growing season, the field was irrigated 12 times, beginning in mid-June and ending in early September, with an irrigation interval of 5–7 days. Each irrigation event applied 300 mm of water (equivalent to 300 L m^−2^). All fertilizers and γ-PGA were applied in six equal splits through drip fertigation, with each split accounting for 16.7% of the total seasonal application rate (compound fertilizer: 47.5 kg ha^−1^; γ-PGA: 0.75 g ha^−1^). The fertigation dates were 17 June, 1 July, 15 July, 29 July, 12 August, and 26 August.

### 2.3. Collection of Cotton Field Soil Samples

The first soil sampling was conducted on 17 August 2023 at the boll-setting stage of cotton, representing the most vigorous growth and development phase. The second sampling was performed on 25 October 2023 during the post-harvest fallow period. At each sampling time, five sampling points were established per plot using a five-point composite sampling method. At each point, five cotton plants with uniform growth were selected, and the root systems with adhering soil were carefully excavated using a sterilized spade within a 15–20 cm radius from the plant base, keeping the root–soil complex as intact as possible. The roots with attached soil were first gently shaken 2–3 times in sterile Ziplock bags to allow large soil clods and bulk soil loosely surrounding the root mass, which were not significantly affected by root exudates, to fall off. The fine soil that remained tightly adhered to the root surface and did not dislodge after gentle shaking (a thin layer approximately 0–2/0–5 mm from the root surface) was considered rhizosphere soil. This layer was brushed off with a sterile brush into sterile bags as the rhizosphere soil sample. After rhizosphere soil was independently collected from each of the five sampling points, the samples from the same plot were pooled into one composite sample, which was then divided into subsamples. One subsample was stored at −80 °C for metagenomic sequencing, and the other subsample was air-dried, ground, and passed through a 0.15 mm stainless steel sieve (100 mesh, Shaoxing Shangyu Huafeng Hardware Instrument Co., Ltd, Shaoxing, China.) for physicochemical property analyses (Table S5).

### 2.4. Determination of Soil Physicochemical Properties

Soil pH was measured using a pH meter at a soil-to-water ratio of 1:5 (*w*/*v*) [22]. Total organic carbon (TOC) was determined by the potassium dichromate oxidation method [23]. Total nitrogen (TN) was measured by the Kjeldahl digestion method [24]. Ammonium nitrogen (NH_4_^+^-N) was extracted with 2 M KCl and determined by the indophenol blue colorimetric method [25]; nitrate nitrogen (NO_3_^−^-N) was measured by the phenol disulfonic acid colorimetric method [26]; and nitrite nitrogen (NO_2_^−^-N) was determined by the N-(1-naphthyl)-ethylenediamine colorimetric method [27].

### 2.5. Soil Metagenomic DNA Extraction, Library Construction, and Sequencing

Total soil DNA was extracted using the TGuide S96 Magnetic Bead Soil/Stool Genomic DNA Extraction Kit (Tiangen Biotech, DP812, Beijing, China). Briefly, 0.25–0.5 g of soil sample was weighed and mixed with buffer SA, buffer SC, and grinding beads, and homogenized using a tissue homogenizer (Tiangen Biotech, TGrinder H24, Beijing, China) at 6 m/s for 30 s with a 30 s interval for 2 cycles. After lysis and magnetic bead purification to remove proteins and impurities, total DNA was eluted with 50–100 μL of elution buffer. DNA concentration and purity were assessed using a Synergy HTX microplate reader (Gene Company Limited, Hong Kong, China) and 1.8% agarose gel electrophoresis.

After passing quality control, the DNA was randomly fragmented, followed by end repair, A-tailing, adapter ligation, fragment size selection, library amplification, and product purification to construct sequencing libraries. The libraries were sequenced on an Illumina NovaSeq 6000 platform (San Diego, CA, USA) using paired-end (PE) sequencing with a read length of 150 bp (PE150).

### 2.6. Metagenomic Bioinformatics Analysis

Data quality control and assembly: Raw reads obtained from sequencing were quality-filtered using Trimmomatic (v0.33) with the parameters LEADING:3, TRAILING:3, SLIDINGWINDOW:50:20, and MINLEN:120 to remove adapter sequences and low-quality reads. After quality control, clean reads were obtained, with an average output of approximately 6.1 Gb per sample (ranging from approximately 5.8 to 6.9 Gb). Clean reads were then de novo-assembled using MEGAHIT (v1.1.2) with default parameters, and contigs shorter than 300 bp were discarded. The assembly quality was evaluated using QUAST v2.3 with default parameters.

Gene prediction and construction of a non-redundant gene catalog: Open reading frames (ORFs) were predicted from the assembled contigs using MetaGeneMark (v3.26) with default parameters. After assembly and ORF prediction, approximately 12,487,954 non-redundant genes were predicted, with a total length of 4,831,939,950 bp, an average length of 386 bp, a maximum length of 24,834 bp, and a minimum length of 102 bp.

The predicted genes from all samples were clustered to remove redundancy using MMseqs2 (version 11-elalc) with a clustering similarity threshold of 95% and a coverage threshold of 90% to construct a non-redundant gene catalog. Clean reads from each sample were then mapped back to the non-redundant gene catalog using Bowtie2 (v2.2.4) to quantify the absolute abundance of each gene in each sample. To eliminate biases caused by gene length and sequencing depth, the abundance of each gene was first normalized using the RPKM method (Reads Per Kilobase per Million mapped reads). Subsequently, the relative percentage abundance of each gene in a given sample was calculated by dividing its RPKM value by the total RPKM value of all genes in that sample, i.e., relative percentage abundance (%) = (RPKM value of the gene/total RPKM value of all genes in the sample) × 100%. All downstream analyses, including diversity analysis, LEfSe differential analysis, and RDA, were directly based on this percentage relative abundance matrix.

Taxonomic and functional annotation: The protein sequences of the non-redundant gene catalog were searched against the NR database using Diamond v0.9.29 with an identity threshold of ≥90%, a coverage threshold of ≥80%, and an e-value cutoff of 1 × 10^−5^. For ambiguous annotations (i.e., multiple homologous matches), the lowest common ancestor (LCA) algorithm was applied to assign taxonomy to the lowest reliable level (hierarchically: kingdom, phylum, class, order, family, genus, species). In addition, sequences were annotated against the eggNOG (v4.0) and KEGG databases for functional annotation using the same alignment parameters.

### 2.7. Statistical Analysis

Soil nutrient data were subjected to one-way analysis of variance (ANOVA), followed by Duncan’s multiple range test for multiple comparisons, with statistical significance set at *p* < 0.05. Repeated-measures two-way ANOVA was used to test the effects of fertilization treatment (NK vs. GT) and sampling time (August vs. October) on soil pH, total organic carbon (TOC), total nitrogen (TN), ammonium nitrogen (NH_4_^+^-N), nitrate nitrogen (NO_3_^−^-N), and nitrite nitrogen (NO_2_^−^-N). For indicators with significant differences, the Bonferroni method was applied for multiple comparison correction.

Before calculating microbial community α-diversity (Shannon and ACE), β-diversity (Binary Jaccard), and performing LEfSe differential analysis, no prevalence filtering, compositional transformation, or rarefaction was applied. All analyses were directly based on the RPKM-normalized relative abundance matrix; the only data transformation was the conversion from RPKM normalization to the percentage relative abundance matrix described in Section 2.6, and all analyses were directly based on this percentage-relative abundance matrix.

Microbial α-diversity indices (Shannon and ACE) were calculated using the diversity and estimateR functions in the vegan package in R v4.3.1. Multiple comparisons among groups were performed using the duncan.test function in the agricolae package for post hoc tests, with significance set at *p* < 0.05.

Microbial β-diversity analysis was based on the Binary Jaccard distance matrix, calculated using the vegdist function in the vegan package, and principal coordinate analysis (PCoA) was performed using the pcoa function in the ape package. Based on the same distance matrix, ANOSIM and PERMANOVA tests were conducted using the anosim and adonis2 functions in the vegan package, each with 999 permutations.

LEfSe analysis was performed using the run_lefse function in the microbiomeMarker package to identify differential species or functional gene biomarkers (LDA score > 3.0, *p* < 0.05).

Redundancy analysis (RDA) was performed using the rda function in the vegan package, and the significance of environmental factors was tested using the envfit function (*p* < 0.05).

Mantel tests were conducted using the mantel function in the vegan package. A Bray–Curtis dissimilarity matrix was calculated from the functional gene relative abundance matrix, and a Euclidean distance matrix was calculated from the Z-score standardized soil physicochemical factors. Pearson correlation was used with 999 permutations. Significance thresholds were set at *p* < 0.05 (** *p* < 0.01, *** *p* < 0.001).

One-way ANOVA and repeated-measures two-way ANOVA were performed using SPSS 26.0 statistical software. Basic plotting and visualization optimization, including composition, color scheme, and layout output, were completed using Origin 2024 Pro software (Table S4).

## 3. Results

### 3.1. Effects of Different Fertilization Treatments on Soil Physicochemical Properties in Cotton Fields

Repeated-measures ANOVA revealed a highly significant interaction effect between fertilization treatment and sampling time on soil physicochemical properties (*p* < 0.01). The pH in the GT treatment was approximately 0.6–0.8 units lower than that in the NK treatment. The total organic carbon (TOC) in the GT treatment increased by approximately 51% from August to October, whereas the TOC in the NK treatment decreased by approximately 13% over the same period. The total nitrogen (TN) in the GT treatment was approximately 23% higher than that in NK in August, and this difference narrowed to approximately 13% in October. In August, the ammonium nitrogen (NH_4_^+^-N) and nitrate nitrogen (NO_3_^−^-N) in the GT treatment were 4.4-fold and 5.9-fold higher than those in NK, respectively. In October, the nitrate nitrogen in the GT treatment decreased to approximately 27% of that in NK. Nitrite nitrogen (NO_2_^−^-N) was generally comparable between the two treatments in August, while both remained at trace levels in October, with the GT treatment approximately 11-fold higher than NK (Table 1 and Table 2, Table S3).

### 3.2. Temporal Dynamics of the Effects of Different Fertilization Treatments on Soil Microbial Community Diversity in Cotton Fields

#### 3.2.1. Alpha-Diversity Analysis of Soil Microbial Communities Under Different Fertilization Treatments

**Figure 1:**
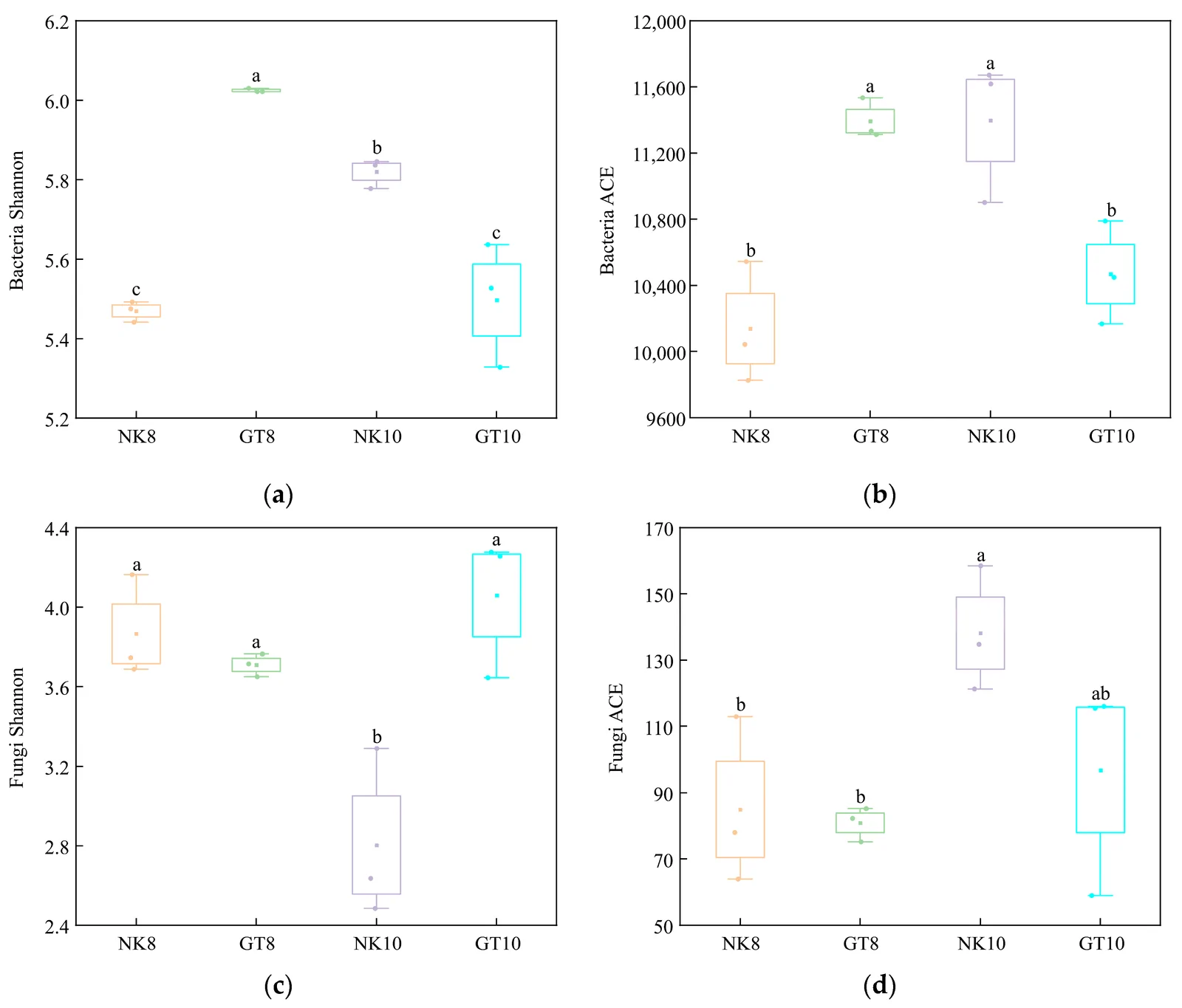

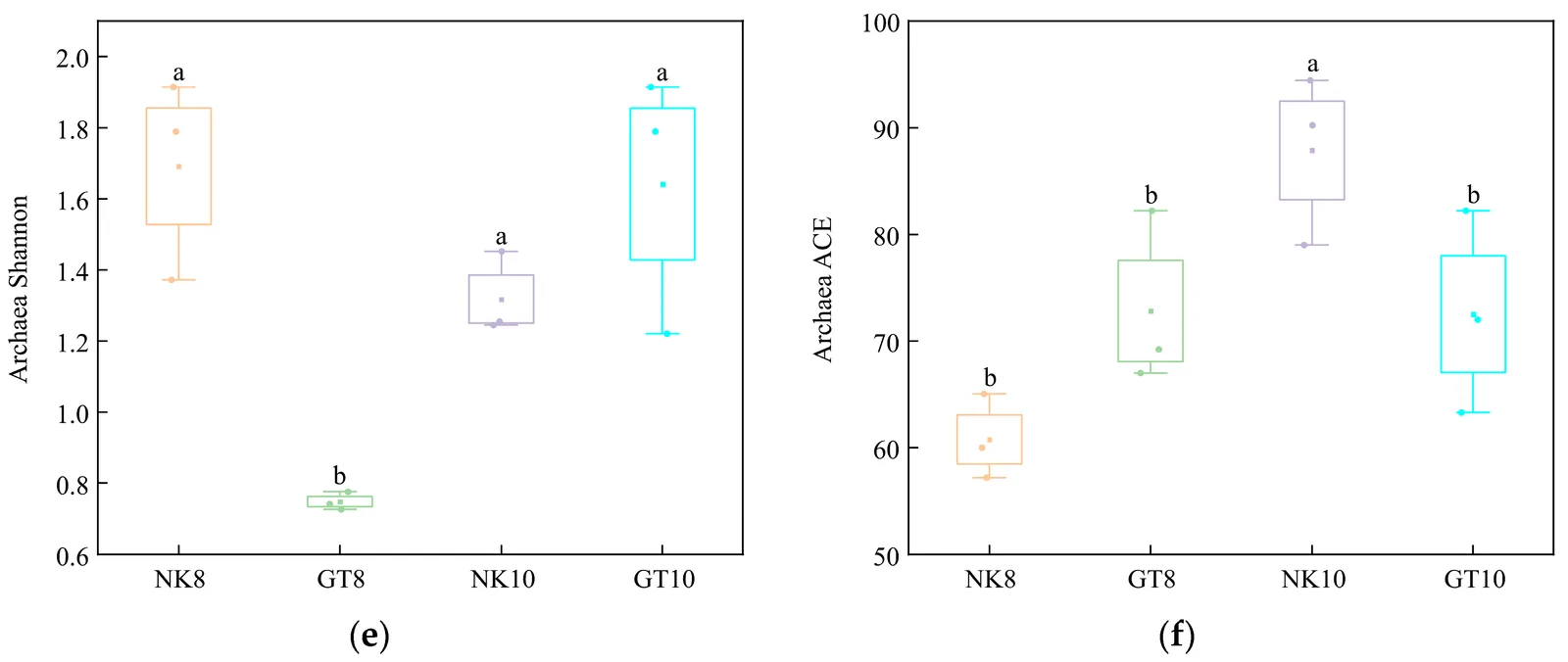
Alpha-diversity indices of soil bacterial, archaeal, and fungal communities under different fertilization treatments. (**a**) Shannon index of bacterial communities; (**b**) ACE richness index of bacterial communities; (**c**) Shannon index of fungal communities; (**d**) ACE richness index of fungal communities; (**e**) Shannon index of archaeal communities; (**f**) ACE richness index of archaeal communities. Data are presented as box plots with individual data points overlaid. The square symbol within each box plot represents the mean, the box indicates the interquartile range (IQR), and the whiskers represent the data range. Different lowercase letters above the box plots indicate significant differences among treatments based on one-way ANOVA followed by Duncan’s multiple range test (*p* < 0.05). NK8, chemical fertilizer alone in August; GT8, γ-PGA combined with chemical fertilizer in August; NK10, chemical fertilizer alone in October; GT10, γ-PGA combined with chemical fertilizer in October.

The results of the alpha-diversity analysis showed that bacterial Shannon and ACE indices were higher in the GT treatment than in the NK treatment in August, whereas both indices were higher in NK than in GT in October. For fungi, the Shannon index was similar between the two treatments in August, while GT exhibited a higher Shannon index than NK in October; the fungal ACE index was comparable between treatments in August, but NK showed a higher ACE index than GT in October. For archaea, the Shannon index in August was higher in NK than in GT, whereas the two treatments reached comparable levels in October; the archaeal ACE index was similar between treatments in August, but NK exhibited a higher ACE index than GT in October. These results indicate that the responses of soil microbial alpha-diversity to different fertilization treatments were highly taxon-specific and exhibited temporal dynamics (Figure 1a–f).

#### 3.2.2. Beta-Diversity Analysis of Soil Microbial Communities Under Different Fertilization Treatments

**Figure 2:**
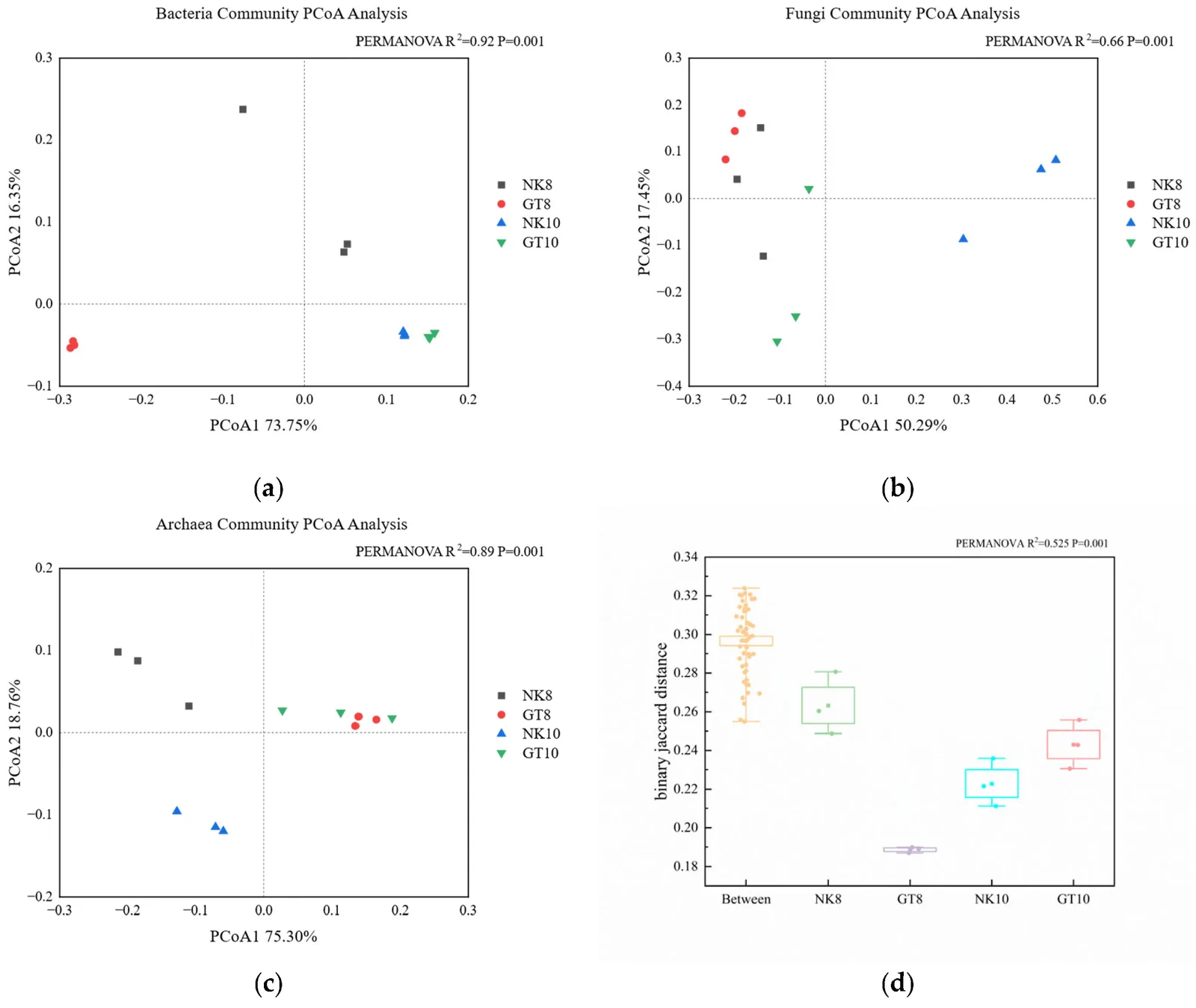
Principal coordinate analysis (PCoA) and analysis of similarities (ANOSIM) of soil microbial communities based on Binary Jaccard distance. (**a**–**c**) PCoA plots of bacterial (**a**), fungal (**b**), and archaeal (**c**) communities. Percentages in parentheses indicate the variation explained by PCoA1 and PCoA2; the R^2^ and *p* values from the PERMANOVA are shown above each panel. (**d**) ANOSIM box plot showing the distribution of distances between groups (Between) and within each treatment group (NK8, GT8, NK10, GT10). Statistical significance was determined by PERMANOVA (R^2^ = 0.525, *p* = 0.001). NK8, chemical fertilizer alone in August; GT8, γ-PGA combined with chemical fertilizer in August; NK10, chemical fertilizer alone in October; GT10, γ-PGA combined with chemical fertilizer in October.

The principal coordinate analysis (PCoA) and PERMANOVA showed that fertilization treatment and sampling time significantly affected the community structure of bacteria, fungi, and archaea (*p* = 0.001). The first two principal coordinate axes (PCoA1 and PCoA2) cumulatively explained 90.10%, 67.74%, and 94.06% of the variation in bacterial, fungal, and archaeal community composition, respectively. The four sample groups (NK8, GT8, NK10, and GT10) exhibited distinct distribution patterns along these axes. For bacterial communities, the NK8 samples were distributed toward the positive end of PCoA2 and GT8 samples toward the negative end of PCoA1, while the NK10 and GT10 samples clustered along the positive end of PCoA1. For fungal communities, both NK8 and GT8 samples in August were distributed toward the negative end of PCoA1; in October, the NK10 samples shifted toward the positive end of PCoA1, whereas the GT10 samples remained on the negative end of PCoA1. For archaeal communities, the GT8 and GT10 samples were distributed along the positive end of PCoA1, whereas the NK8 and NK10 samples were located along the negative end of PCoA1. These results indicate that fertilization treatment and sampling time exerted divergent effects on the spatial patterns of the three microbial community types (Figure 2a–c).

ANOSIM and PERMANOVA based on Jaccard distance further confirmed that fertilization treatment and sampling time significantly influenced soil microbial community structure (*p* = 0.001). Between-group distances exceeded within-group distances. The GT8 group exhibited the lowest within-group distance and NK8 the highest, while NK10 and GT10 showed intermediate values (Figure 2d).

### 3.3. Compositional Characteristics of Microbial Community Structure Under Different Fertilization Treatments

To further elucidate the patterns underlying the beta-diversity changes, the compositional characteristics of bacterial, fungal, and archaeal communities were analyzed at the phylum and genus levels (Table S1).

**Figure 3:**
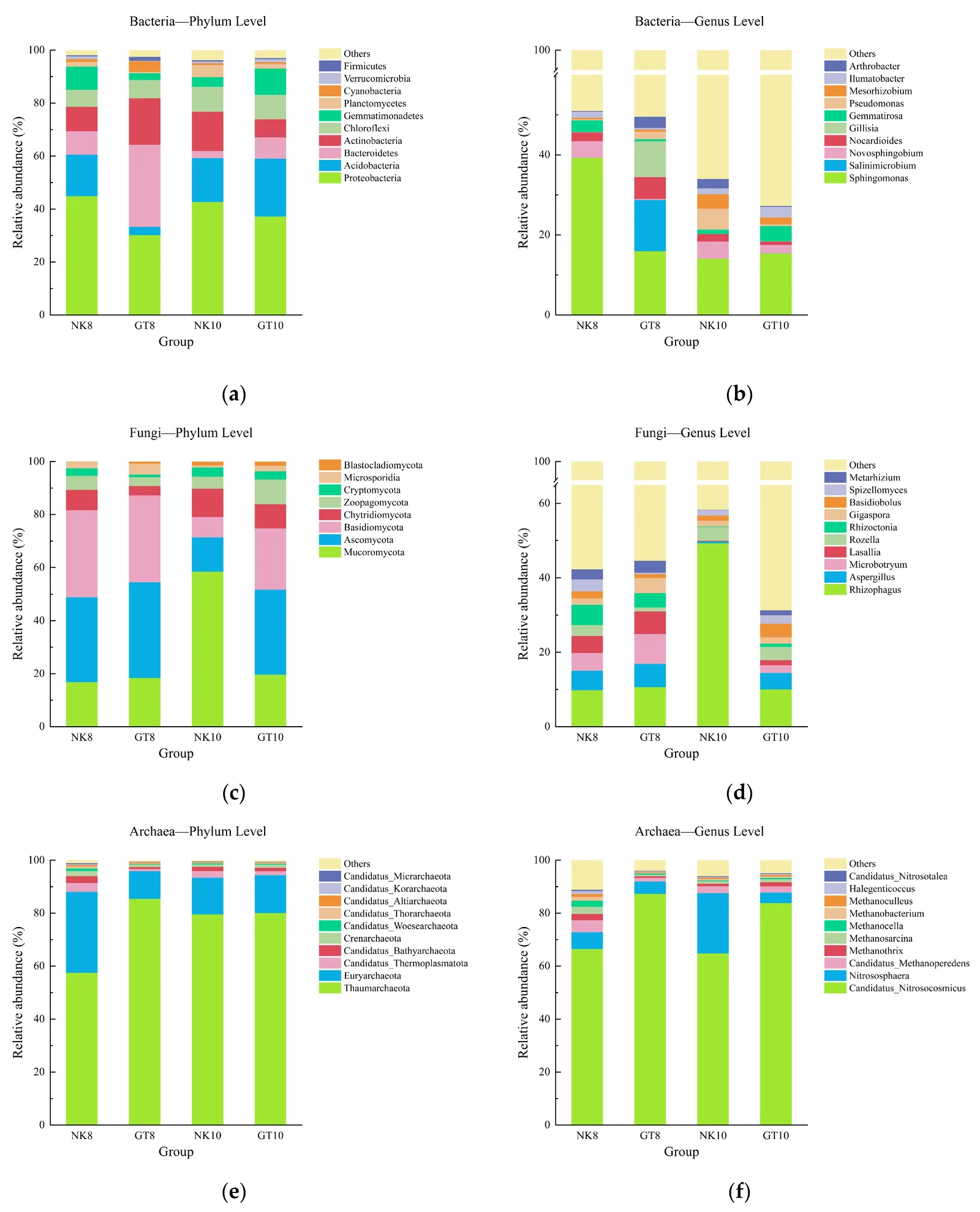
Relative abundances of soil microbial communities at the phylum and genus levels under different fertilization treatments. (**a**) Bacterial—Phylum Level; (**b**) Bacterial—Genus Level; (**c**) Archaeal—Phylum Level; (**d**) Archaeal—Genus Level; (**e**) Fungal—Phylum Level; (**f**) Fungal—Genus Level (top 10). The y-axis represents relative abundance (%). NK8, chemical fertilizer alone in August; GT8, γ-PGA combined with chemical fertilizer in August; NK10, chemical fertilizer alone in October; GT10, γ-PGA combined with chemical fertilizer in October.

In the bacterial community, *Proteobacteria* consistently dominated under the chemical fertilizer alone (NK) treatment, with relative abundances exceeding 40% in both August and October. In contrast, the γ-PGA combined with chemical fertilizer (GT) treatment altered the dominant bacterial taxa pattern. In the GT treatment in August, *Bacteroidetes* (30.9%) and *Actinobacteria* (17.6%) exhibited higher relative abundances than in the NK treatment, while the proportion of *Proteobacteria* decreased to 30.2%. In October, the relative abundances of *Acidobacteria* (21.8%) and *Gemmatimonadetes* (10.0%) in the GT treatment were higher than those observed in August. At the genus level, the relative abundances of *Salinimicrobium* (12.8%), *Gillisia* (8.9%), and *Arthrobacter* (2.7%) increased markedly in the GT treatment in August, whereas *Sphingomonas* (14.1%) and *Pseudomonas* (5.3%) were the dominant taxa in the NK treatment in October (Figure 3a,b).

In the fungal community, the relative abundance of *Mucoromycota* in the NK treatment reached 58.5% in October, surpassing those of *Ascomycota* and *Basidiomycota*. In the same period, the GT treatment maintained *Ascomycota* (32.1%) and *Basidiomycota* (23.1%) as the dominant phyla, with *Mucoromycota* accounting for 19.6%. At the genus level, the relative abundance of *Rhizophagus* reached 49.3% in the NK treatment in October, compared with 10.0% in the GT treatment. In contrast, the proportions of *Basidiobolus* and *Rozella* were higher in the GT treatment than in the NK treatment (Figure 3c,d).

In the archaeal community, the relative abundance of *Thaumarchaeota* in the GT treatment in August was 85.4%, which was higher than that in the NK treatment (57.5%). By October, the relative abundances of *Thaumarchaeota* in the GT and NK treatments converged, reaching 80.1% and 79.5%, respectively. At the genus level, the relative abundance of *Candidatus Nitrosocosmicus* in the GT treatment in August (87.3%) was higher than that in the NK treatment (66.4%). In October, the relative abundance of *Nitrososphaera* was 22.8% in the NK treatment, whereas it accounted for only 3.9% in the GT treatment, where *Candidatus Nitrosocosmicus* remained the dominant genus (Figure 3e,f).

### 3.4. Functional Differences in Soil Microbial Communities Under Different Fertilization Treatments

**Figure 4:**
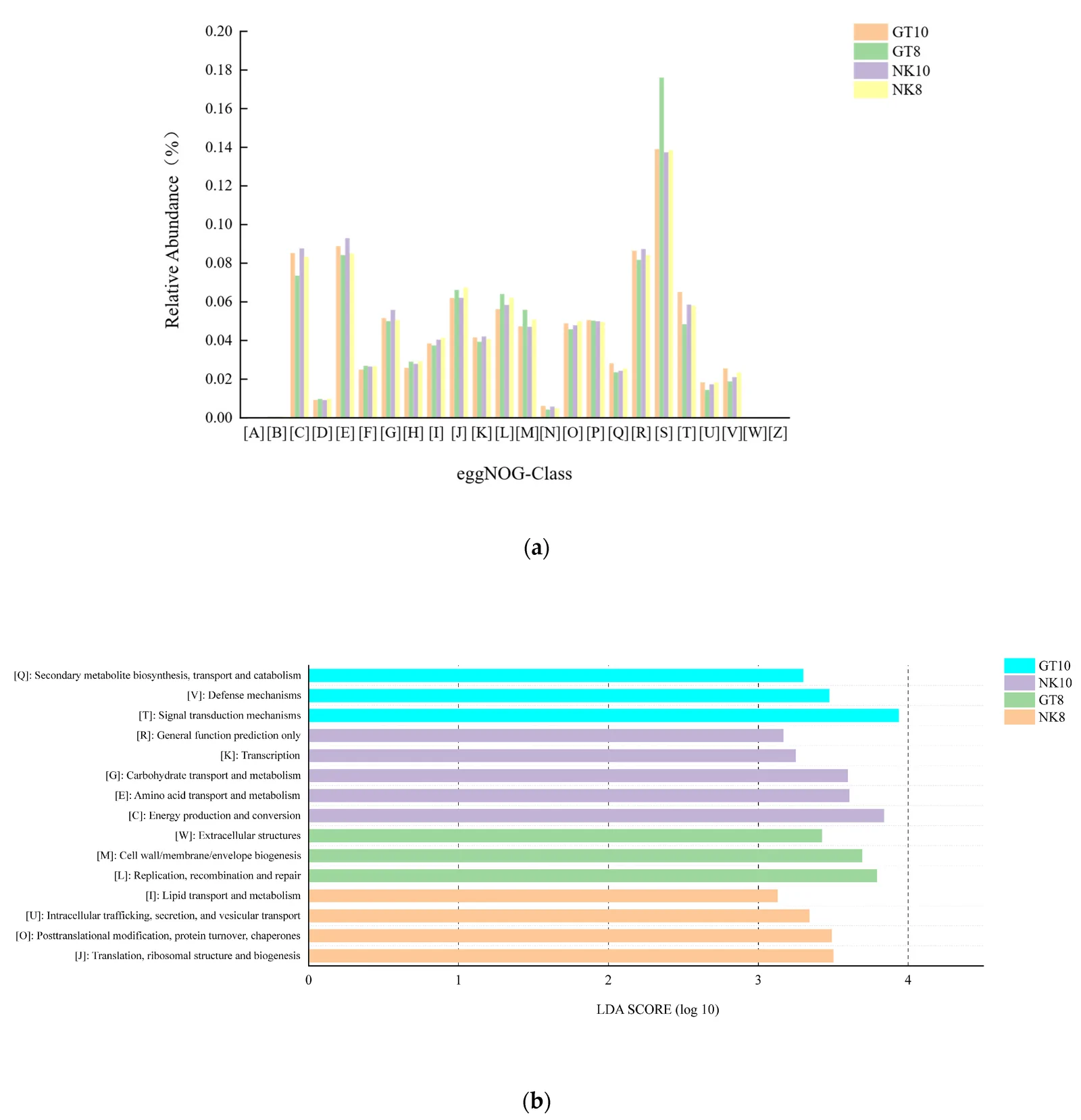
(**a**) Relative abundances of eggNOG secondary functional categories in soil microbial communities under different fertilization treatments. The x-axis indicates functional category codes based on the EggNOG database, which are classified into four major functional groups: Information storage and processing, including [A]: RNA processing and modification, [B]: Chromatin structure and dynamics, [J]: Translation, ribosomal structure and biogenesis, [K]: Transcription, [L]: Replication, recombination and repair; Cellular processes and signaling, including [D]: Cell cycle control, cell division, chromosome partitioning, [M]: Cell wall/membrane/envelope biogenesis, [N]: Cell motility, [O]: Post-translational modification, protein turnover, chaperones, [T]: Signal transduction mechanisms, [U]: Intracellular trafficking, secretion, and vesicular transport, [V]: Defense mechanisms, [W]: Extracellular structures, [Z]: Cytoskeleton; Metabolism, including [C]: Energy production and conversion, [E]: Amino acid transport and metabolism, [F]: Nucleotide transport and metabolism, [G]: Carbohydrate transport and metabolism, [H]: Coenzyme transport and metabolism, [I]: Lipid transport and metabolism, [P]: Inorganic ion transport and metabolism, [Q]: Secondary metabolite biosynthesis, transport and catabolism; Poorly characterized, including [R]: General function prediction only, [S]: Function unknown. The y-axis represents relative abundance (%), and different colors (orange, green, purple, yellow) indicate the corresponding functional modules in each treatment group (NK8, GT8, NK10, GT10). (**b**) Significantly differentially enriched functional biomarkers among treatment groups identified by LEfSe analysis (LDA score > 3.0, *p* < 0.05). The length of the bars represents the LDA score (log10), and different colors (orange, green, purple, cyan) indicate functional modules with significantly higher relative abundances in the corresponding treatment group (NK8, GT8, NK10, GT10). The letter codes in parentheses correspond to the orthologous functional cluster classifications in the EggNOG database.

Functional annotation based on the eggNOG database revealed that metabolic functions dominated the functional categories across all samples (Table S2). With respect to the relative abundance distribution of specific functional subcategories, at the August boll-setting stage, the GT treatment exhibited higher relative abundances than NK in functions related to [M]: Cell wall/membrane/envelope biogenesis, [W]: Extracellular structures, and [L]: Replication, recombination and repair. In contrast, the NK treatment showed higher relative abundances in functions related to [J]: Translation, ribosomal structure and biogenesis, [O]: Post-translational modification, protein turnover, chaperones, [I]: Lipid transport and metabolism, and [U]: Intracellular trafficking, secretion, and vesicular transport. At the October fallow period, the functional abundance patterns diverged markedly. The NK treatment surpassed the GT treatment in the categories of [C]: Energy production and conversion, [G]: Carbohydrate transport and metabolism, [E]: Amino acid transport and metabolism, and [K]: Transcription. Conversely, the GT treatment showed higher relative abundances than NK in functions related to [T]: Signal transduction mechanisms, [V]: Defense mechanisms, and [Q]: Secondary metabolite biosynthesis, transport and catabolism. Further LEfSe analysis showed that in August, GT8 was significantly enriched in [L]: Replication, recombination and repair, [W]: Extracellular structures, and [M]: Cell wall/membrane/envelope biogenesis, whereas NK8 was significantly enriched in [O]: Post-translational modification, protein turnover, chaperones, [I]: Lipid transport and metabolism, [U]: Intracellular trafficking, secretion, and vesicular transport, and [J]: Translation, ribosomal structure and biogenesis. In October, GT10 was significantly enriched in [Q]: Secondary metabolite biosynthesis, transport and catabolism, [T]: Signal transduction mechanisms, and [V]: Defense mechanisms, whereas NK10 was significantly enriched in [E]: Amino acid transport and metabolism, [K]: Transcription, [G]: Carbohydrate transport and metabolism, and [C]: Energy production and conversion (Figure 4a,b).

### 3.5. Redundancy Analysis of Soil Microbial Functions and Physicochemical Properties Under Different Fertilization Treatments

**Figure 5:**
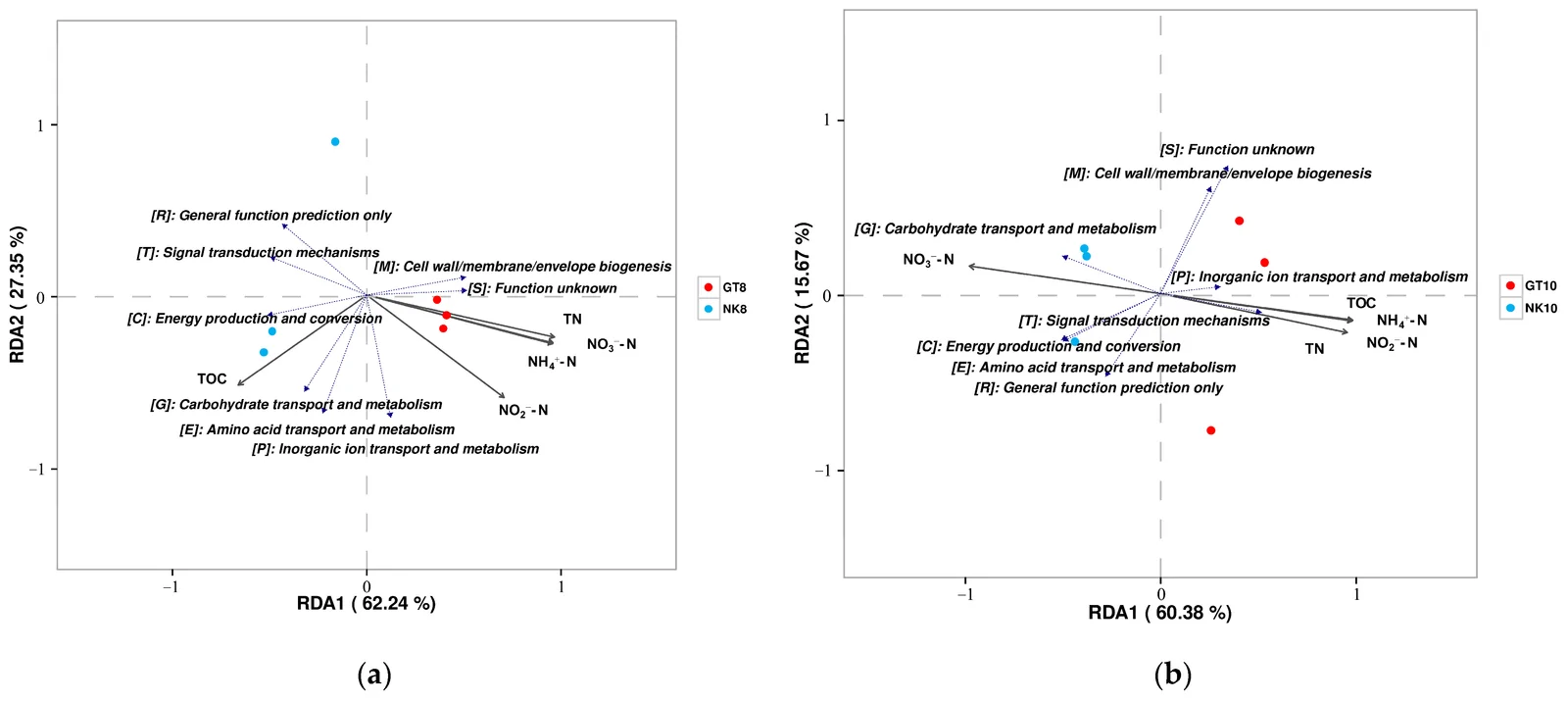
Redundancy analysis (RDA) of soil microbial community functions and soil physicochemical properties. (**a**) RDA of microbial functional profiles and soil physicochemical factors at the August sampling (NK8 vs. GT8); (**b**) RDA of microbial functional profiles and soil physicochemical factors at the October sampling (NK10 vs. GT10). NK, chemical fertilizer alone; GT, chemical fertilizer combined with γ-PGA; 8 and 10 denote the August and October sampling times, respectively. Blue dashed arrows indicate functional genes annotated by the eggNOG database.

Redundancy analysis (RDA) showed that the first two axes explained 89.59% of the total variation in August and 76.05% in October. In August, NK8 and GT8 samples were clearly separated along the positive and negative directions of the RDA1 axis, respectively. The arrows of TOC and [E]: Amino acid transport and metabolism, [C]: Energy production and conversion, [G]: Carbohydrate transport and metabolism, and [T]: Signal transduction mechanisms were oriented in the same direction as the NK8 sample cluster, indicating positive correlations. In contrast, the arrows of TN, NH_4_^+^-N, NO_3_^−^-N, and NO_2_^−^-N, together with [P]: Inorganic ion transport and metabolism and [M]: Cell wall/membrane/envelope biogenesis, were oriented in the same direction as the GT8 sample cluster, indicating positive correlations. In October, the NK10 and GT10 samples were also clearly separated along the positive and negative directions of the RDA1 axis. Unlike the pattern observed in August, the arrow of NO_3_^−^-N pointed toward the negative direction of the RDA1 axis, whereas the arrow of TOC pointed toward the positive direction. Among functional genes, [T]: Signal transduction mechanisms pointed toward the positive direction of the RDA1 axis (Figure 5a,b).

### 3.6. Mantel Analysis of Soil Microbial Functions and Physicochemical Properties Under Different Fertilization Treatments

**Figure 6:**
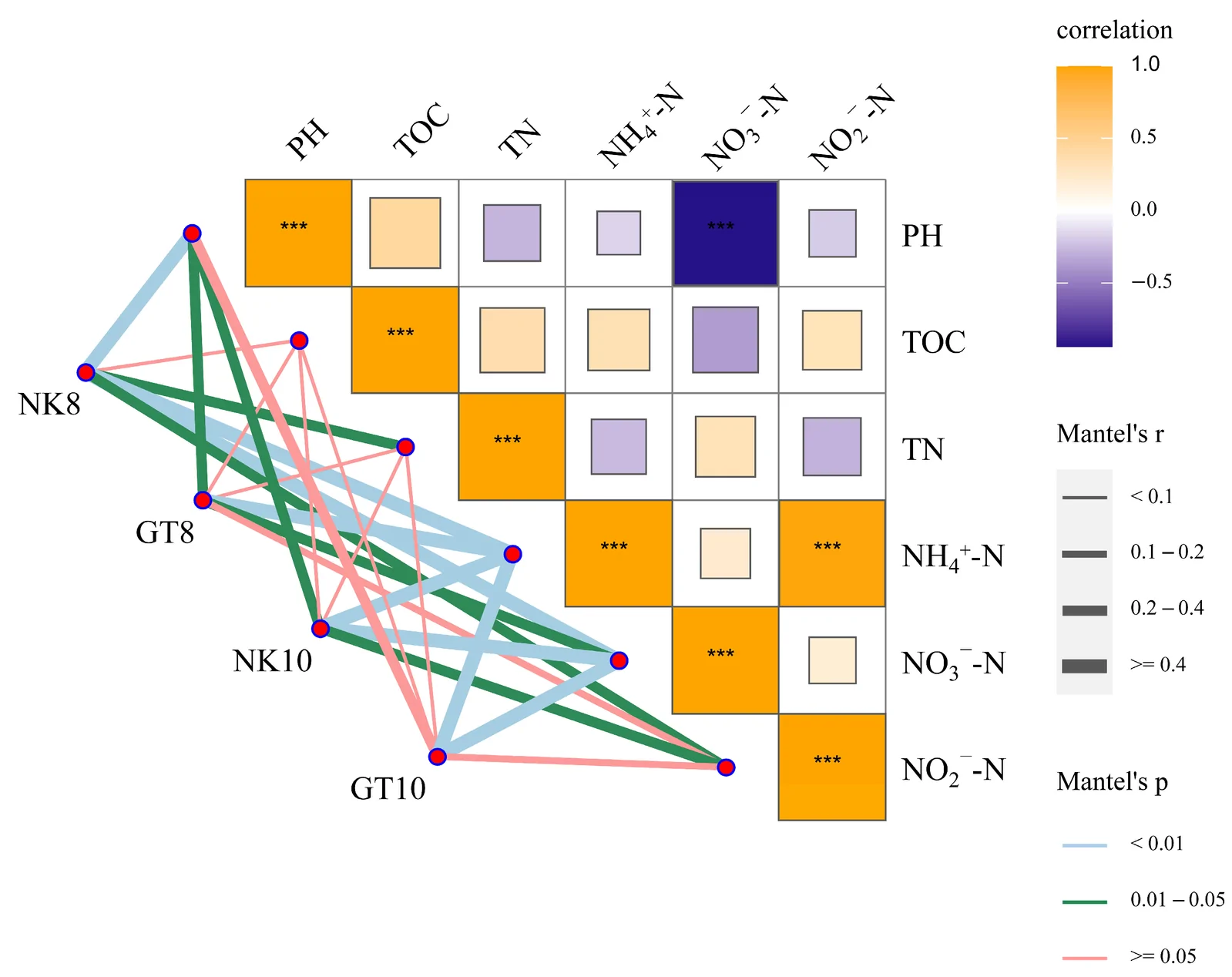
Heatmap and Mantel test network integrating environmental factors. In the heatmap, cell color represents the Pearson correlation coefficient between environmental factors; asterisks indicate statistical significance (*** *p* < 0.001, ** *p* < 0.01, * *p* < 0.05). In the Mantel test network, line color denotes significance (*p* value), and line thickness denotes the strength of the Mantel test (r statistic). NK8, chemical fertilizer alone in August; GT8, γ-PGA combined with chemical fertilizer in August; NK10, chemical fertilizer alone in October; GT10, γ-PGA combined with chemical fertilizer in October.

The Mantel test analysis revealed that NH_4_^+^-N exerted a highly significant driving effect on all four treatment groups (Mantel’s r ≥ 0.75, *p* < 0.01). Regarding pH, in August, pH was highly significantly correlated with the microbial functional community of NK8 (r = 0.81, *p* < 0.01) and also significantly correlated with GT8 (r = 0.23, *p* < 0.05). In October, pH was significantly correlated with NK10 (r = 0.33, *p* < 0.05), but showed no significant correlation with GT10 (*p* > 0.05). NO_3_^−^-N exhibited relatively strong associations with the microbial functional communities of NK8, NK10, and GT10 (r ≥ 0.41, *p* < 0.05 or *p* < 0.01), whereas its association with GT8 was comparatively weaker. TN was only associated with the microbial functional community of NK8 (r = 0.32, *p* < 0.05), whereas TOC showed no significant correlation with any treatment group (*p* > 0.05). Pearson correlation analysis among environmental factors showed that pH was highly significantly negatively correlated with NO_3_^−^-N, while NO_3_^−^-N was highly significantly positively correlated with NH_4_^+^-N. Overall, the variation in microbial functional communities was primarily regulated by nitrogen species, particularly NH_4_^+^-N and NO_3_^−^-N (Figure 6).

## 4. Discussion

### 4.1. Effects of γ-Polyglutamic Acid Application on Soil Physicochemical Properties in Cotton Fields

Soil physicochemical properties are core components of the rhizosphere microenvironment. The forms of nutrient occurrence and acid–base characteristics directly determine soil nutrient supply capacity and serve as the habitat basis for microbial communities, and are therefore fundamental indicators for evaluating the soil effects of exogenous regulatory substances [28,29]. In this study, the repeated-measures two-way ANOVA showed that fertilization treatment, sampling time, and their interaction had extremely significant effects on soil pH, TOC, TN, NH_4_^+^-N, NO_3_^−^-N, and NO_2_^−^-N (*p* < 0.001) (Table 1). This indicates that the regulation of the rhizosphere soil environment by γ-PGA is not a simple linear ameliorative effect; rather, the direction and magnitude of its effects shift with crop growth progression. At the August boll-setting stage, the most vigorous growth phase of cotton, rhizosphere metabolism was highly active. Compared with the NK treatment, the GT treatment significantly reduced rhizosphere pH and increased NH_4_^+^-N, NO_3_^−^-N, and TN contents by 339.3%, 491.4%, and 23.0%, respectively. The core mechanism lies in the abundant free carboxyl groups along the γ-PGA molecular chain, which can adsorb and retain ammonium ions through cation exchange [13]. The increases in NO_3_^−^-N and TN contents were consistent with the trends observed by Tu et al. in their study on γ-PGA-amended soils [30]. At this stage, no significant difference in TOC was detected between the two treatments, primarily because photosynthates are preferentially allocated to aboveground development during the growing season, resulting in a time-lag effect in carbon input from root residues and exudates [31]. Moreover, the carbon input from γ-PGA itself is limited and insufficient to alter the size of the soil TOC pool in the short term. By the October post-harvest fallow period, the input of root exudates ceased. Under the NK treatment, soil mineralization continued, leading to substantial NO_3_^−^-N accumulation up to 66.85 mg/kg, accompanied by a decline in TOC, thereby forming a high-nitrate nutrient regime. Previous studies have shown that nitrate accumulated during the non-growing season is the main source of leaching loss, and soil organic nitrogen mineralization during the fallow period can contribute 48.2% of nitrate leaching [32,33]. The high NO_3_^−^-N concentration detected in the NK treatment therefore indicates a potential subsequent leaching risk. In contrast, under the GT treatment, γ-PGA continuously exerted its functions in ammonium retention and organic carbon sequestration [13,34]. TOC in GT was 70.6% higher than in NK, and nitrogen was stored predominantly as NH_4_^+^-N, which effectively restricted the substrate supply for nitrification and greatly reduced NO_3_^−^-N content, thereby establishing a stable rhizosphere microenvironment conducive to nutrient retention (Table 2). This temporal differentiation in soil nitrogen pool forms and acid–base conditions constituted the core environmental filtering basis for the subsequent assembly and functional transformation of rhizosphere microbial communities.

### 4.2. Effects of γ-Polyglutamic Acid Application on Soil Microbial Community Structure in Cotton Fields

Soil microbial community diversity is a key indicator of rhizosphere microecological structural stability and functional potential [35], and changes in species richness and evenness directly reflect the regulatory effects of exogenous fertilization on the soil microenvironment [36]. This study showed that the regulatory effects of γ-PGA combined with chemical fertilizer differed markedly among bacterial, fungal, and archaeal communities, and these differences exhibited distinct patterns across cotton growth stages. The alpha-diversity results indicated that in August, at the boll-setting stage, the bacterial Shannon and ACE indices were significantly higher in the GT treatment than in NK, as the high availability of nitrogen substrates supported the rapid proliferation of copiotrophic taxa [37]. In October, during the fallow period, bacterial diversity in the GT treatment decreased, whereas NK showed higher diversity, corresponding to the reduced input of plant-derived carbon after harvest and the gradual dominance of oligotrophic taxa, consistent with the classical succession of microbial communities from r-strategy to K-strategy [38]. Fungal diversity did not differ significantly between treatments in August, when arbuscular mycorrhizal fungi and saprotrophic fungi coexisted under the active root symbiotic environment, maintaining a balanced community structure [39]. By October, the NK treatment showed a pattern of increased richness but decreased evenness, reflecting the explosive proliferation of r-strategist taxa such as *Mucoromycota* utilizing residual root substrates [40]. In contrast, the fungal Shannon index remained at a relatively high level in the GT treatment, with a moderate increase in the ACE index, and no overexpansion of any single taxon was observed. This suggests that γ-PGA application may buffer the ecological disturbance caused by carbon source interruption after cotton harvest, suppressing the excessive proliferation of r-strategist saprotrophic fungi such as *Mucoromycota* and thereby maintaining fungal community structural stability, which is consistent with the findings of Yin et al. in their study on the rhizosphere microbial environment of γ-PGA-treated maize [17]. Archaeal alpha-diversity was consistently regulated by soil ammonium nitrogen concentration. In August, under the high NH_4_^+^-N environment, the archaeal community in the GT treatment shifted toward the single ammonium-tolerant taxon *Candidatus Nitrosocosmicus*, with a significant decrease in evenness. In October, as NH_4_^+^-N concentration decreased, archaeal evenness increased in the GT treatment. In contrast, under NK, ammonium nitrogen was depleted, and the proportion of the low-ammonium-tolerant taxon *Nitrososphaera* increased, while community evenness remained persistently low, reflecting the high sensitivity of ammonia-oxidizing archaea to nitrogen substrate forms [41,42]. PCoA and PERMANOVA based on Binary Jaccard distance showed that bacterial, fungal, and archaeal communities were all significantly differentiated along the sampling time axis, and the differences between the two treatments within each period were highly significant (*p* = 0.001). The treatment explained 0.92 and 0.89 of the variation in bacterial and archaeal communities, respectively, indicating that γ-PGA application had statistically robust effects on community restructuring (Figure 2a–c). ANOSIM within-group distance analysis showed that the mean within-group distance of GT8 (0.189) was lower than that of NK8 (0.263), reflecting that γ-PGA reduced the community spatial heterogeneity by homogenizing the rhizosphere microenvironment during the vigorous growing season. By the fallow period, the within-group distance of GT10 (0.243) increased to a level higher than that of NK10 (0.223) and also higher than that of GT8 (Figure 2d), indicating that the homogenizing effect of γ-PGA on community structure weakened as the growth stage progressed.

### 4.3. Effects of γ-Polyglutamic Acid Application on Soil Microbial Functional Potential in Cotton Fields

Soil microbial functional potential is the core link between community structure and soil ecological processes, and the composition of metabolic pathways directly reflects the potential capacity for rhizosphere nutrient cycling and environmental adaptation [43]. Based on functional annotation against the eggNOG database [44], metabolism dominated the functional categories across all samples. An analysis of relative abundance at the class level showed that, in August, the GT treatment exhibited significantly higher abundances of functions related to [M]: Cell wall/membrane/envelope biogenesis and [W]: Extracellular structures, whereas the NK8 treatment showed a clear advantage in [O]: Post-translational modification, protein turnover, chaperones, [I]: Lipid transport and metabolism, [U]: Intracellular trafficking, secretion, and vesicular transport, and [J]: Translation, ribosomal structure and biogenesis, reflecting the structural expansion and resource acquisition tendencies of the microbial community during the vigorous growing season. By the October fallow period, the functional distributions diverged significantly. NK10 remained dominated by primary metabolic functions such as [C]: Energy production and conversion, [E]: Amino acid transport and metabolism, and [G]: Carbohydrate transport and metabolism. In contrast, GT10 showed higher relative abundances of [T]: Signal transduction mechanisms, [V]: Defense mechanisms, and [Q]: Secondary metabolite biosynthesis, transport and catabolism. This reshaped functional abundance pattern, characterized by attenuated primary metabolism and enhanced defense-related functions, precisely indicated an active shift in the rhizosphere microbial ecological strategy from resource acquisition to resource conservation (Figure 4a). LEfSe differential analysis further demonstrated that γ-PGA application not only altered the taxonomic composition of the rhizosphere microbiome but also drove significant changes in community functional potential across growth stages. These changes were highly consistent with shifts in microbial community composition, collectively reflecting the adaptive responses of the microbial community to rhizosphere environmental remodeling. At the August boll-setting stage, the GT treatment was significantly enriched in [M]: Cell wall/membrane/envelope biogenesis, [W]: Extracellular structures, and [L]: Replication, recombination and repair, possibly indicating an active proliferation state of the microbial community under this treatment. The NK treatment was mainly enriched in [J]: Translation, ribosomal structure and biogenesis, [O]: Post-translational modification, protein turnover, chaperones, [I]: Lipid transport and metabolism, and [U]: Intracellular trafficking, secretion, and vesicular transport, suggesting that its functional potential was more concentrated on intracellular material processing and turnover. During the October fallow period, the functional enrichment patterns of the two treatments diverged markedly. The NK treatment was significantly enriched in [C]: Energy production and conversion, [G]: Carbohydrate transport and metabolism, [E]: Amino acid transport and metabolism, and [K]: Transcription, corresponding to the substrate decomposition and nutrient transformation processes sustained by residual soil nitrate. In contrast, the GT treatment did not show significant enrichment in primary metabolism-related functions; instead, [T]: Signal transduction mechanisms, [V]: Defense mechanisms, and [Q]: Secondary metabolite biosynthesis, transport and catabolism were significantly enriched. This suggests that the GT microbial community may have shifted from a rapid growth strategy to a conservative survival strategy oriented toward environmental adaptation and homeostasis, which is consistent with the dominance of oligotrophic taxa in the community composition. The RDA results showed that the first two axes explained 76.05% of the total variation in October. The NK10 samples were positively correlated with residual nitrate and remained functionally associated with resource acquisition metabolic pathways. The GT10 samples were jointly driven by total organic carbon and total nitrogen, with functional potential shifting toward [T]: Signal transduction mechanisms, [P]: Inorganic ion transport and metabolism, and [M]: Cell wall/membrane/envelope biogenesis, fully consistent with the functional characteristics of an oligotrophic conservative strategy [45,46]. It should be noted that the relative abundances of functional genes obtained from metagenomic sequencing in this study only reflect the functional potential of the community rather than the in situ metabolic activity of microorganisms or actual nutrient transformation fluxes. The actual metabolic state, gene expression levels, and elemental transformation rates of microorganisms during the fallow period still require further verification through approaches such as metatranscriptomics and stable isotope tracing [47].

### 4.4. Relationships Between Microbial Functional Profiles and Cotton Field Soil Physicochemical Properties

The Mantel test was used to analyze the correlations between microbial functional gene composition and soil physicochemical factors [48]. The results showed that γ-PGA co-application altered the relationships between these factors and microbial functions, shifting from simultaneous association with multiple factors to a predominant association with ammonium nitrogen. This finding is consistent with the observed changes in soil nutrients and microbial community structure. At the August boll-setting stage, NH_4_^+^-N exerted highly significant driving effects on the microbial functional communities in both the NK and GT treatments. The functional community in the NK treatment was also significantly correlated with pH, NO_3_^−^-N, and TN, indicating that, under chemical fertilizer alone, the microbial functional community was jointly influenced by soil pH and multiple nitrogen nutrients. In the GT treatment in August, the functional community exhibited a significant but weak association with pH, a relatively weak association with NO_3_^−^-N, and no significant association with TN, whereas NH_4_^+^-N showed the strongest driving effect. This suggests that γ-PGA may stabilize soil pH and retain ammonium nitrogen through chelation [13], thereby reducing the interference of pH and nitrate fluctuations on the microbial functional community and making ammonium nitrogen the primary driving factor. During the October fallow period, the functional community in the NK treatment remained significantly correlated with pH, NH_4_^+^-N, and NO_3_^−^-N, indicating that its microbial functional community was still jointly influenced by soil pH and multiple nitrogen forms. In the GT treatment, NH_4_^+^-N remained highly significantly correlated, and its association with NO_3_^−^-N was stronger than in August; however, no significant correlations were observed with pH, TN, or TOC. This indicates that during the fallow period, the microbial functional community in the GT treatment was primarily regulated by nitrogen forms such as ammonium and nitrate, with less influence from non-nitrogen factors such as pH. Overall, the differences in microbial functional communities among treatments were mainly driven by nitrogen forms, particularly NH_4_^+^-N and NO_3_^−^-N. Compared with the NK treatment, the GT treatment weakened the associations with pH and TN to some extent, and the dominant role of nitrogen forms in shaping microbial functional communities became more prominent (Figure 6).

**Figure 7:**
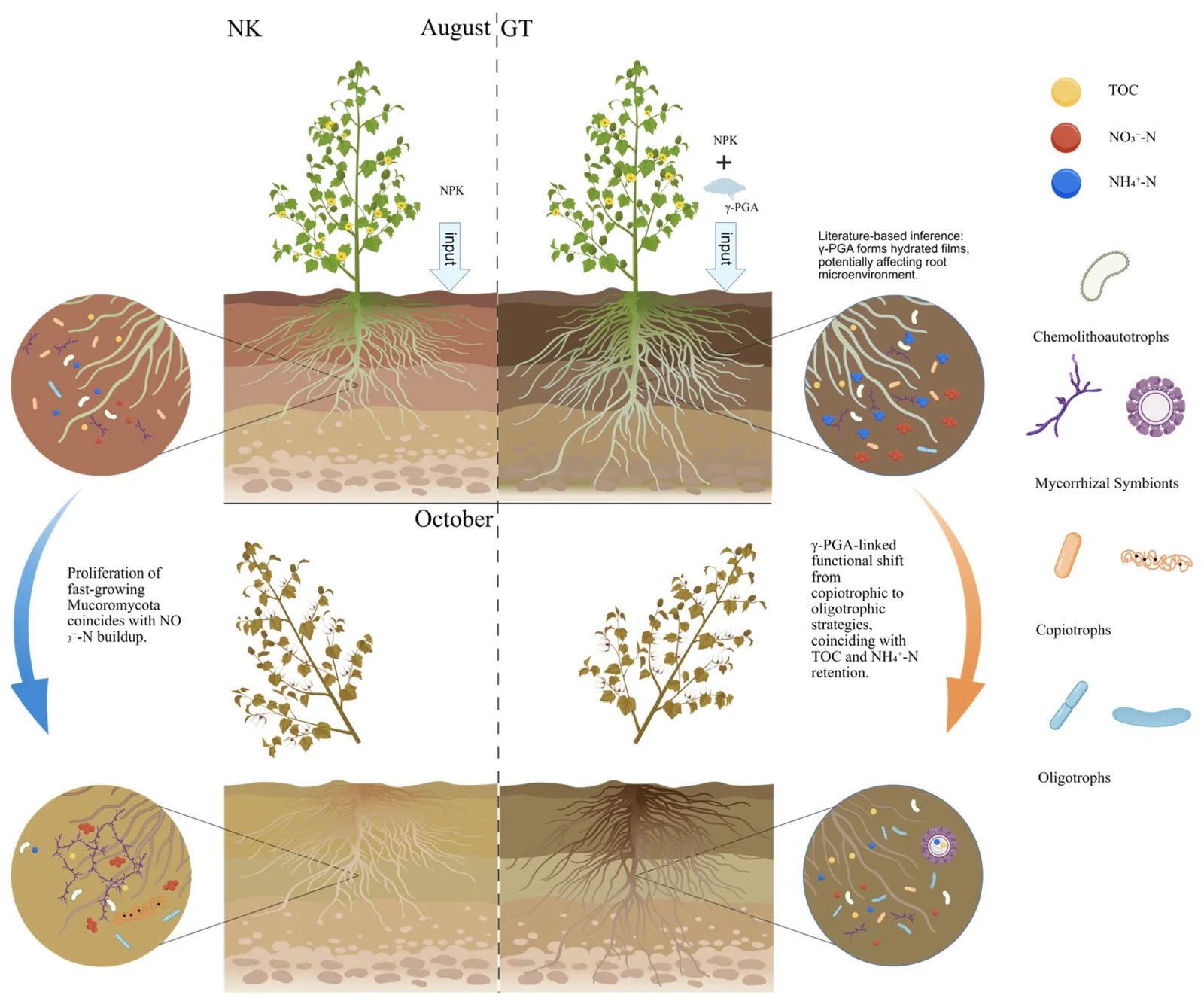
Hypothetical conceptual model of γ-PGA application driving rhizosphere microbial community dynamics. The model was constructed based on measured physicochemical and metagenomic data from August (boll-setting stage) and October (fallow period). Conventional fertilization (NK) pathway (left): In October, TOC declines and NO_3_^−^-N accumulates; massive proliferation of *Mucoromycota* coincides with nitrate accumulation, and functions are dominated by primary metabolism, representing a “resource acquisition” strategy with potential leaching risk. γ-PGA combined with chemical fertilizer (GT) pathway (right): In August, nitrogen is mobilized and symbiotic microbial groups are enriched; in October, TOC and NH_4_^+^-N are significantly retained, driving a shift in functional strategy from copiotrophic to oligotrophic (as indicated in the figure), thereby achieving nutrient retention. The top bubbles “γ-PGA forms a hydration film,” mycelial networks, dormant vesicles, and other microscopic physical structures shown in the figure are inferred from the literature and were not directly verified by microscopic imaging or activity measurements in this study; the exact mechanisms require future investigation combining microscopic imaging, metatranscriptomics, and isotope tracing. Legend: yellow = TOC; red = NO_3_^−^-N; blue = NH_4_^+^-N.

Based on the above correlation analysis results, the changes in microbial communities under the two fertilization regimes were summarized (Figure 7). Under conventional fertilization (NK), the microbial community in August was influenced by multiple environmental factors and was dominated by rapid growth. By October, nitrate nitrogen accumulated in the soil, and fungi represented by *Mucoromycota* proliferated extensively, resulting in a simplified community structure and a tendency for nutrient loss. In contrast, under γ-PGA combined with chemical fertilizer (GT), the microbial community in August grew under a stable supply of ammonium nitrogen, with copiotrophic bacteria being dominant. By October, soil organic carbon and ammonium nitrogen were maintained at relatively high levels, oligotrophic bacteria became the dominant taxa, and the fungal community structure remained even, which was conducive to nutrient retention.

## 5. Conclusions

This study investigated the effects of γ-polyglutamic acid (γ-PGA) combined with chemical fertilizer on soil physicochemical properties, microbial communities, and functional potential in cotton fields at different growth stages. The results showed that γ-PGA co-application increased rhizosphere soil ammonium nitrogen (NH_4_^+^-N) and organic carbon contents, altered the nutrient status of the rhizosphere soil, buffered the impact of post-harvest carbon source interruption on fungal community structure, and drove bacterial community succession from copiotrophic taxa toward oligotrophic taxa, while the archaeal community shifted toward ammonium-preferring taxa. Meanwhile, microbial functional potential shifted from resource acquisition during the vigorous growing season to conservative maintenance during the fallow period. In conclusion, the co-application of γ-PGA with chemical fertilizer is a fertilization strategy that can maintain soil microbial community stability and reduce carbon and nitrogen nutrient losses during the fallow period, providing a basis for rational fertilization in arid cotton fields.

## Figures and Tables

**Table 1:** Results of repeated-measures two-way ANOVA testing the effects of fertilization treatment, sampling time, and their interaction on soil physicochemical properties.

	df	PH		TOC		TN		NH_4_^+^-N		NO_3_^−^-N		NO_2_^−^-N	
		*F*	*p*	*F*	*p*	*F*	*p*	*F*	*p*	*F*	*p*	*F*	*p*
Time	1	31.24	=0.005	1207.39	<0.001	559.44	<0.001	112,378.47	<0.001	660.58	<0.001	211,288.08	<0.001
Treatment	1	188.18	<0.001	705.33	<0.001	4005.12	<0.001	84,127.75	<0.001	3103.69	<0.001	673.77	<0.001
Interaction	1	422.29	<0.001	3464.20	<0.001	21.87	=0.009	51,337.40	<0.001	37,094.33	<0.001	400.74	<0.001

Note: df, degrees of freedom; *F*, *F* statistic of the repeated-measures two-way ANOVA; *p*, probability value of significance. Time, sampling time (August vs. October); Treatment, fertilization treatment (NK vs. GT); Interaction, the interaction effect between time and treatment. TOC, total organic carbon; TN, total nitrogen; NH_4_^+^-N, ammonium nitrogen; NO_3_^−^-N, nitrate nitrogen; NO_2_^−^-N, nitrite nitrogen. *p* < 0.05 indicates significant difference; *p* < 0.01 indicates highly significant difference.

**Table 2:** Effects of different fertilization treatments on soil physicochemical properties at the boll-setting stage (August) and fallow period (October).

Treatment	pH	TOC (g·kg^−1^)	TN (g·kg^−1^)	NH_4_^+^-N (mg·kg^−1^)	NO_3_^−^-N (mg·kg^−1^)	NO_2_^−^-N (mg·kg^−1^)
NK8	8.14 ± 0.01 a	16.43 ± 0.46 b	1.52 ± 0.02 c	20.13 ± 0.31 b	14.62 ± 0.20 d	8.43 ± 0.05 b
GT8	7.40 ± 0.01 d	16.12 ± 0.12 b	1.87 ± 0.01 b	88.44 ± 0.33 a	86.47 ± 0.78 a	8.53 ± 0.04 a
NK10	7.61 ± 0.08 c	14.31 ± 0.19 c	1.87 ± 0.02 b	6.58 ± 0.05 d	66.85 ± 0.45 b	0.08 ± 0.01 d
GT10	7.70 ± 0.01 b	24.41 ± 0.07 a	2.11 ± 0.02 a	18.38 ± 0.02 c	18.15 ± 0.10 c	0.88 ± 0.03 c

Note: Values are presented as means ± standard deviation (*n* = 3). Different lowercase letters within the same column indicate significant differences among treatments (*p* < 0.05, Duncan’s multiple range test). NK, chemical fertilizer alone; GT, γ-PGA combined with chemical fertilizer; 8, August (boll-setting stage); 10, October (fallow period). TOC, total organic carbon; TN, total nitrogen; NH_4_^+^-N, ammonium nitrogen; NO_3_^−^-N, nitrate nitrogen; NO_2_^−^-N, nitrite nitrogen.

## Data Availability

The taxonomic and functional annotation results (Tables S1 and S2) and soil physicochemical data (Table S3) are provided as Supplementary Materials. All specific software versions, R packages, function names, and parameters used for the analyses are detailed in Section 2 (Materials and Methods) of the main text, ensuring the transparency and reproducibility of the analytical process. The raw metagenomic sequencing reads are not publicly available due to a related patent application but are available from the corresponding author upon reasonable request.

## Supplementary Materials

The following supporting information can be downloaded at https://www.mdpi.com/article/10.3390/microorganisms14091905/s1: Table S1. Microbial taxonomic classification table; Table S2. Functional classification table; Table S3. Soil physicochemical properties table. Table S4. Experimental Design, Field Management, and Analytical Protocols for the Cotton Rhizosphere Metagenomic Study (2023 Season). Table S5. Schedule of Agronomic Practices, Irrigation, Fertilization, and Rhizosphere Soil Sampling Across the Cotton Growing Season (2023).
